# Consequences of interplant trait variation for canopy light absorption and photosynthesis

**DOI:** 10.3389/fpls.2023.1012718

**Published:** 2023-01-20

**Authors:** Maarten van der Meer, Hyeran Lee, Pieter H. B. de Visser, Ep Heuvelink, Leo F. M. Marcelis

**Affiliations:** ^1^ Horticulture and Product Physiology, Wageningen University, Wageningen, Netherlands; ^2^ Business Unit Greenhouse Horticulture, Wageningen Research, Wageningen, Netherlands

**Keywords:** functional–structural plant model (FSPM), plant-to-plant variation, interplant variation, tomato, photosynthesis, light absorption

## Abstract

Plant-to-plant variation (interplant variation) may play an important role in determining individual plant and whole canopy performance, where interplant variation in architecture and photosynthesis traits has direct effects on light absorption and photosynthesis. We aimed to quantify the importance of observed interplant variation on both whole-plant and canopy light absorption and photosynthesis. Plant architecture was measured in two experiments with fruiting tomato crops (*Solanum lycopersicum*) grown in glasshouses in the Netherlands, in week 16 (Exp. 1) or week 19 (Exp. 2) after transplanting. Experiment 1 included four cultivars grown under three supplementary lighting treatments, and Experiment 2 included two different row orientations. Measured interplant variations of the architectural traits, namely, internode length, leaf area, petiole angle, and leaflet angle, as well as literature data on the interplant variation of the photosynthesis traits alpha, *J*_max28_, and *V*_cmax28_, were incorporated in a static functional–structural plant model (FSPM). The FSPM was used to analyze light absorption and net photosynthesis of whole plants in response to interplant variation in architectural and photosynthesis traits. Depending on the trait, introducing interplant variation in architecture and photosynthesis traits in a functional–structural plant model did not affect or negatively affected canopy light absorption and net photosynthesis compared with the reference model without interplant variation. Introducing interplant variation of architectural and photosynthesis traits in FSPM results in a more realistic simulation of variation of plants within a canopy. Furthermore, it can improve the accuracy of simulation of canopy light interception and photosynthesis although these effects at the canopy level are relatively small (*<*4% for light absorption and<7% for net photosynthesis).

## Introduction

Plant-to-plant variation (interplant variation) may play an important role in determining individual plant and whole canopy performance (Westerband et al., 2021), where interplant variation in architecture and photosynthesis traits has direct effects on light absorption and photosynthesis (Sarlikioti et al., 2011a). Interplant variation has been studied for a longer time in ecology, greenhouse horticulture, and field crops, where uniformity is of importance in greenhouse horticulture and field crops. The fruits of greenhouse cucumber show a changing demand in assimilates over time and abortion of individual fruits (Marcelis, 1992). These characteristics result in non-uniform fruit growth and are also observed in other reproductive and indeterminate crops such as bell pepper and tomato (Heuvelink, 1996; Wubs et al., 2009b). Differences in whole-plant fruit growth have been linked to environmental factors such as light intensity, photoperiod, CO_2_ concentration, temperature, relative air humidity, water and nutrient supply (Marcelis, 1993; Wubs et al., 2009a; Pettersen et al., 2010), and canopy architecture (Chen et al., 2014).

Functional–structural plant models (FSPMs) explore and integrate relationships between a plant’s structure and processes that underlie its growth and development (Louarn and Song, 2020). These models have been developed for a wide range of crops and for many purposes (Louarn and Song, 2020) including studies on the effects of plant architecture on light absorption and photosynthesis in a canopy (e.g., Cieslak et al., 2010; Buck-Sorlin et al., 2011; Wiechers et al., 2011; Chen et al., 2014; van der Meer et al., 2021). Models have also been used to study interplant variation in organ growth and development, where the variation may originate from competition for carbon (e.g., Luquet et al., 2006; Kang et al., 2011) or from different forms of signaling, in which signals may be internal to the plant, external, or an integration of both (Fournier et al., 2005; Alban et al., 2008; Evers et al., 2011).

In the last years, there has been a considerable improvement in carbon-driven growth through sink strength modeling, and several successful models have been developed that predict the mean plant and allow for the incorporation of interplant variation such as Greenlab (de Reffye et al., 2021) and Ecomeristem (Larue et al., 2019). The main functions for determining variability are branching, tillering time, and senescence. The importance of including interplant variation in architectural and photosynthesis traits for modeling light absorption and photosynthesis can be structurally assessed by model simulations but has yet received minimal attention. Zhu et al. (2015) investigated this topic in a maize and soybean mixture and showed that the inclusion of interplant variation can result in enhanced light capture.

The aim of this study was to determine the importance of interplant variation in architectural and leaf photosynthetic traits on canopy light absorption and net photosynthesis. For this, we used measured interplant variation of architectural and leaf photosynthetic traits in fruiting tomato plants and used that as input in a static functional–structural plant model to simulate the consequences of interplant variation on canopy light absorption and net photosynthesis.

## Material and methods

### Architectural data acquisition

Data on plant architecture were collected from two separate experiments with tomato crops (*Solanum lycopersicum*) grown in glasshouses in Bleiswijk, The Netherlands (Exp. 1), and in Wageningen, The Netherlands (Exp. 2; described by van der Meer et al., 2021).

#### Experiment 1—cultivar × lighting

Four tomato cultivars (cv. ‘Foundation,’ ‘Progression,’ ‘Extension.’ and ‘9112’) were sown on 28 August 2015 and transplanted into glasshouse compartments (6 October 2015) when the first truss emerged, having a split stem and 8-10 leaves per stem. Three treatments with supplementary lighting to natural light were applied to the four tomato cultivars: 1) HPS top lighting (110 µmol m^−2^ s^−1^) + LED intracanopy lighting (53 µmol m^−2^ s^−1^) at two heights (2 and 2.5 m), 2) LED top lighting (110 µmol m^−2^ s^−1^) + LED intracanopy lighting (53 µmol m^−2^ s^−1^) at two heights (2 and 2.5 m), and 3) LED top lighting (110 µmol m^−2^ s^−1^) + two intracanopy lighting (53 µmol m^−2^ s^−1^) at two heights (2.5 and 3 m). Top lighting was provided using either HPS or LED lamps (Philips Greenpower, 95% red and 5% blue, Signify, Eindhoven, The Netherlands) at a height of 4.75 m above the floor. Intracanopy lighting was provided by Philips Greenpower interlighting LED lamps (95% red and 5% blue, Signify, Eindhoven, The Netherlands). The lower and upper leaves were on average at 1.5 and 3.5 m height above the floor respectively, whereas the rockwool slab was at 0.9 m height of the floor. On 14 October 2015, lamps were turned on, gradually increasing the duration of lighting to a daily maximum of 19 h (0:00 h until 1 h after sunset). When the outside solar radiation exceeded 600 W m^−2^, the lamps were switched off. The mean daily temperature, relative humidity, and CO_2_ concentration for the three treatments were 20.2°C, 80%, and 666 ppm (1); 20.3°C, 82%, and 695 ppm (2); and 20.3°C, 83%, and 702 ppm (3), respectively, while the outside global radiation was on average 3.2 MJ m^−2^ day^−1^. Light transmission through the greenhouse cover was 64%.

**Figure 1:**
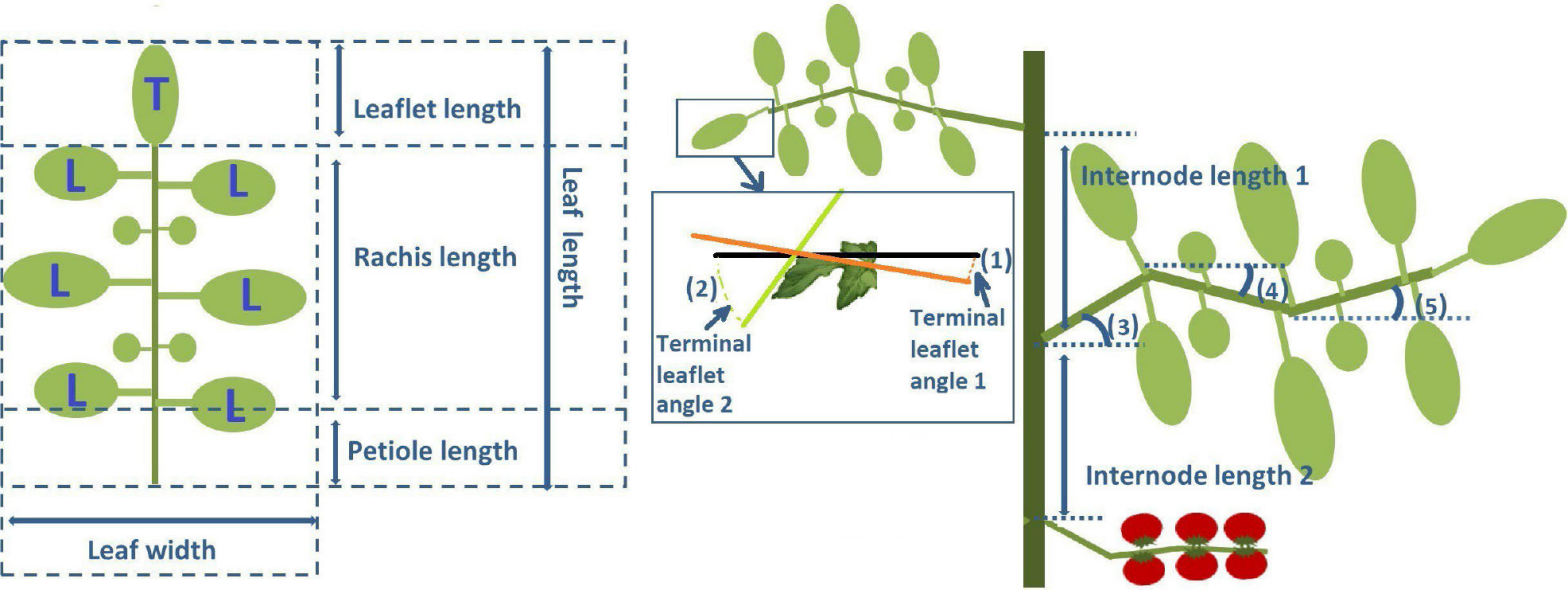
Diagram of measured plant architecture at the phytomer level. On the left side, a leaflet with a terminal leaflet (T) and six lateral leaflets (L). On the right-hand side, a stem section with two phytomers each containing an internode, petiole, and leaflet. Indicated are (1, 2) a zoomed-in terminal leaflet showing the measurement of terminal leaflet angle 1 (from the base of the leaflet to the middle point) and terminal leaflet angle 2 (from the tip of the leaflet to the middle point) relative to the horizon (3, 4, 5) petiole angle, first rachis bend and second rachis bend which are all measured relative to the horizon, and internode lengths 1 and 2 of the two phytomers.


Plant architecture was measured 16 weeks after transplanting (25 - 29 January 2016) on five plants in each of the 12 treatments (4 cultivars × 3 light treatments), for a total of 60 plants. Leaf length and width, internode length, petiole angle, and the first and second rachis bending angles were measured at every phytomer rank (**Figure 1**). Leaf length was measured from the petiole to the tip of the terminal leaflet. Leaf width was measured between the tips of the two longest leaflets. The measured internode length was the distance between the insertion points of two consecutive plant organs (leaves or truss). The petiole angle and the first and second rachis bend angles were measured relative to the horizon. Rachis bend angles were measured at the first and second big leaflet pairs (**Figure 1**). Stem density on the day of measuring was 3.1 stems m^−2^. Leaf area was estimated from the leaf length and leaf width for the second experiment according to the formula

(1) $$Leaf\;area=A*{\left(leaf\;length*leaf\;width\right)}^{B}$$

where *A* and *B* are dimensionless parameters where values for each cultivar are taken from the data of Exp. 2 (for values, see **Supplementary Table S1**).

#### Experiment 2—row orientation

Five-week-old tomato plants (cv. ‘Capriccia’) were transplanted into greenhouse compartments in either a north–south or east–west row orientation on 8 March 2016. The plants were grown until 20 July 2016. A nutrient solution (EC: 2.8 and pH: 5.5; see **Supporting Information** **Table S2**) was provided daily in a frequency matching solar radiation. Side stems were removed weekly, and from 13 April onward, the bottom three leaves were removed every week. From 25 April, the plants reached the high wire at a height of 3.3 m, after which they were lowered weekly. The plants were grown in double rows, with a distance of 50 cm between the single rows. Trusses were pruned to 6 fruits per truss. The two glasshouse compartments were oriented –24° north–south. The architecture of 12 plants was measured in both the north–south and east–west row orientations 19 weeks after transplanting (20 July 2016), for a total of 24 plants. For this study, architectural measurements on each phytomere of internode length, leaf length, and leaf area were used. Stem density on the day of measuring was 4.4 stems m^−2^. Temperature, relative humidity, and CO_2_ concentration averaged over the whole growth period were 20.1°C and 20.0°C, 75% and 74%, and 509 and 518 ppm for the compartments with north–south and east–west row orientations, respectively. The outside global radiation was on average 15.5 MJ m^−2^ day^−1^. Light transmission through the greenhouse cover was 62%. For more details on this experiment, see van der Meer et al. (2021).

### Modeling

The tomato functional–structural plant model used has been described and validated by van der Meer et al. (2021) and Schipper et al. (2022) with reparameterization of some architectural parameters based on the measurements in Experiments 1 and 2. This model was developed on the GroIMP platform (Kniemeyer, 2008) and consists of architectural, light, and photosynthesis modules. The essentials of each module are briefly explained, while for a more elaborate explanation, we refer to van der Meer et al. (2021).

#### Architectural module

**Figure 2:**
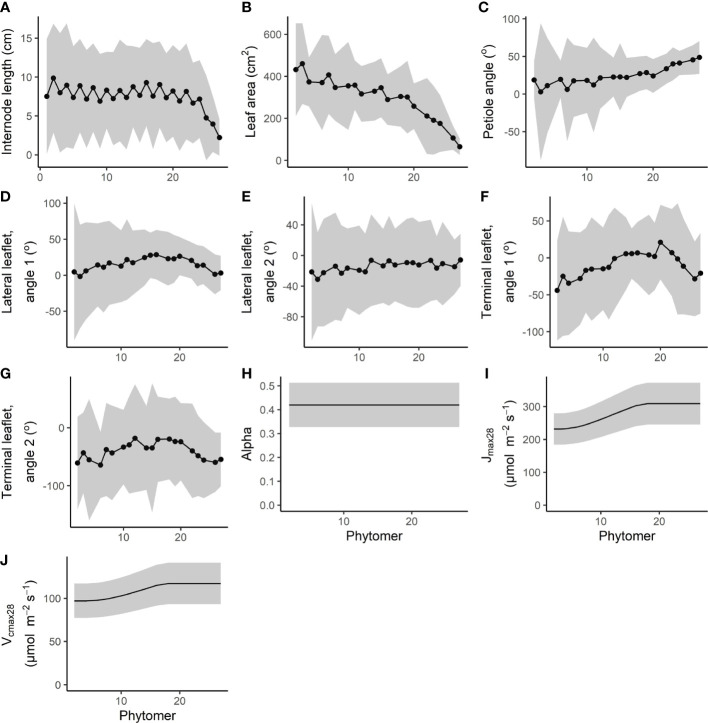
Measured interplant variation of **(A–G)** architectural traits combined from the two glasshouse experiments (for details on how the architectural parameters were measured, see **Figure 1**), as well as **(H–J)** interplant variation in photosynthesis traits. Alpha, *J*_max28_, and *V*_cmax28_ mean values with variation were taken from Qian et al. (2012) who performed measurements on two heights. Mean values for *J*_max28_ and *V*_cmax28_ were assumed equal from the first to the ninth top from the leaf and then decreased exponentially as a result of lower light levels from the 10th leaf from the top to the lowest leaf of the plant. Variation in photosynthesis parameters was kept relational to the mean. Data are represented as the mean ±2 times the standard deviation. Every fourth phytomer bears a truss instead of a leaf and is therefore presented as missing data, with the exception of internode length.

The plants were grown in double rows, with a distance of 50 cm between the single rows. The distance between the mids of two adjacent double rows was 160 cm, resulting in a path of 62 cm (distance between the outer leaves of each row). The distance between plants in each single row was 50 cm. Architecture trait reference values were estimated encompassing all measurements on the 84 plants total (60 from Exp. 1 and 24 from Exp. 2) of both experiments and were used to construct a reference plant (**Figure 2**). This reference plant was copied 216 times (6 double rows with 36 plants per double row) to construct a reference canopy, where each plant was rotated (0° to 360°, drawn at random) relative to its neighbor, and these orientations were then fixed during all simulations. Taking measurement data together from the two experiments and five genotypes increased the range where trait variability was derived from. This was done as follows. Firstly, for each trait, a mean and variance were calculated for each phytomer rank of each genotype for each treatment in each experiment. Afterward, for every trait for every phytomer rank, the calculated means and variances were averaged. In this way, one mean value with standard deviation was calculated for each phytomer for leaf area, stem length, and leaf length with measurements of both experiments and for rachis bend and leaflet angles with data from the first experiment. The number of leaflets was 11 (6 lateral and 1 terminal leaflet), and the leaf area was then distributed across the 11 leaflets on each leaf according to an empirical allometric relationship. The area of the composite leaf was distributed over the five leaflet pairs and the terminal leaflet for each leaf in the same manner, using the following fractions (from proximal to distal leaflet pairs): 0.1296, 0.0188, 0.1516, 0.0281, and 0.0948 and the terminal leaflet with 0.1541 (from Exp. 2). The length of the petiole was 35% of the total leaf length, and at that point, the first leaflet pair was attached.

#### Photosynthesis module

An adapted Farquhar–von Caemmerer–Berry model (FvCB model) was used to simulate photosynthesis (Farquhar et al., 1980; Qian et al., 2012). Mean values with variation for alpha, *J*_max28_, and *V*_cmax28_ were taken from a set of measurements at the middle and top height of the canopy as described in Qian et al. (2012). *J*_max_ and *V*_cmax_ were assumed to decrease in an exponential way from the 10*^th^* leaf from the top to the oldest leaf as a result of a decrease in light; *J*_max_ decreased from 309 to 232 µmol m^−2^ s^−1^ and *V*_cmax_ from 117 to 97 µmol m^−2^ s^−1^. Variation in photosynthesis parameters was introduced as a fraction of the mean. This assumption of exponential decrease was taken since there was no support for any other relationship. Aside from this, model simulations have shown that overestimation of photosynthesis when acclimation of photosynthetic parameters with height in the canopy is not considered is minimal when the only light source is the sun (van Ieperen and Trouwborst, 2008).

#### Light module

The light in the 3D scene was simulated in hourly time steps with a ray tracer, called the Flux Light Model, provided by GroIMP. The Flux Light Model was described in detail by Henke and Buck-Sorlin (2017). This ray tracer was based on an inversed path tracer with a Monte Carlo pseudo-random number generator as in Veach (1997). The number of rays was 750 million and the recursion depth was 10; these numbers were chosen such that a further increase would not improve simulation results (see **Supplementary Table S3**). For diffuse radiation, the assumption was made of an overcast sky, where light sources were located according to azimuth at every 7.5° and zenith at every 15°. The remainder fraction of light was direct light and modeled as a point source of directional light, arriving from the hourly solar position (model from Goudriaan and van Laar, 1994). Light transmission through the greenhouse cover was 60%.

### Model scenarios

One reference canopy was created, which contained 216 plants. Each plant was rotated (0° to 360°, drawn at random) relative to its neighbor, and for the reference canopy, this was the only source of variation. The rotation of the plants relative to their neighbors was kept the same for every model simulation such that any differences in light absorption and net photosynthesis are the result of including interplant variation of architectural and/or photosynthesis traits. No collision avoidance of the leaves was computed, since the leaves of neighboring plants in reality do not show distinct avoidance mechanisms and extremely intertwine. Every model simulation was run with fixed environmental variables.

Model simulations were run to study light absorption and photosynthesis for a full-grown tomato canopy under differing architectural and photosynthesis trait variations. Trait variation was introduced by drawing from the normal distribution for each trait from these measured data and was independent of other ranks in the same plant. To ensure equal leaf area and internode length between different model scenarios, for each phytomer value drawn, there was a plant on the mirrored other side of the canopy with a value that deviated the opposite from the mean (see **Supporting Information**-**Figure S1**). Ten scenarios with or without interplant variation for specific traits were compared: 1) reference, 2) internode length, 3) leaf area, 4) petiole angle, 5) leaflet angle, 6) full architecture (internode length, leaf area, petiole angle, and leaflet angle combined), 7) alpha, 8) *J*_max_ and *V*_cmax_, 9) full photosynthesis (alpha, *J*_max_, and *V*_cmax_ combined), and 10) everything combined (full architecture and full photosynthesis).

Planting densities were 1.5, 2.4, and 3.3 plants m^−2^, with LAI 1.75, 2.80, and 3.85, respectively. The model was run with hourly time steps at a fixed CO_2_ concentration of 600 ppm and a temperature of 23°C. The model was run for summer and winter solstices (21 June, 21 December) at latitude 52° with both days 0% and 77% direct light. Each model simulation was repeated 5 times with a different value for the random number generator, which randomizes any parameter with a random component in it, i.e., the values drawn from the normal distribution as well as the light model ray tracer. Differences between 5 repetitions were on maximum 2.2% for canopy light absorption, 6.4% for average net photosynthesis, 26.5% for the coefficient of variation of plant light absorption, and 23.0% for the coefficient of variation of plant net photosynthesis. Calculations were performed for the center 12 plants in the center two double rows (24 plants in total).

## Results

### Architectural variation in tomato canopies

The coefficient of variation (CV) of internode length and leaf area remained fairly constant from bottom to top with a maximum of 60% for internode length and 43% for leaf area (**Figure 2**). For the petiole angle, the standard deviation was rather large at the bottom with a maximum of 46°, compared to a minimum of 8° at the top. For leaflet angles of the lateral and terminal leaflets, the ranges were similar between a minimum of 11° found at the top leaves and a maximum of 56° at the bottom of the canopy.

### Interplant variation reduces canopy light absorption and net photosynthesis

**Figure 3:**
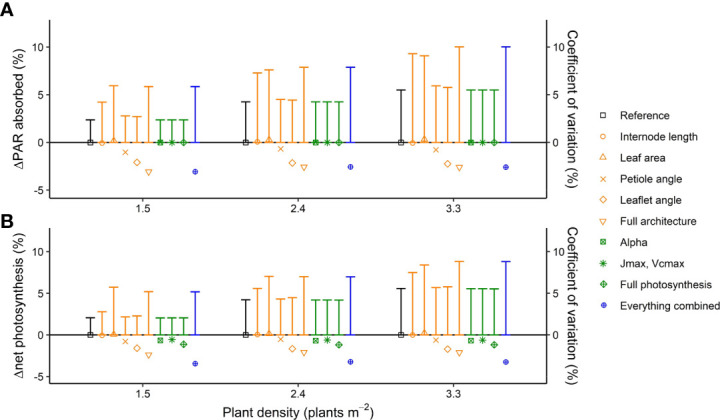
Simulated effects of interplant variation in architectural and photosynthesis traits on **(A)** canopy light absorption (symbols) and the coefficient of variation in plant light absorption (vertical bars) and **(B)** net photosynthesis (symbols) and the coefficient of variation in photosynthesis (vertical bars) at three planting densities. Canopy light absorption and plant net photosynthesis are presented as % change compared to the reference where the rotation of plants relative to their neighbors (0 to 360◦, drawn at random) was the only source of variation. The rotation of the plants relative to their neighbors was kept the same for every model simulation such that any differences in light absorption and net photosynthesis are the result of including interplant variation of architectural and/or photosynthesis traits. Data are the averaged values of all hourly time steps in a day of 24 plants (centre 12 plants of the centre two double rows) from five repetitions (simulations with each a different random number generator for the light model and drawings from the normal distribution), at two fractions of direct light (0 and 0.77) and two solstices summer and winter (DOY 171 and 356), resulting in a total of 480 plants.

Introducing interplant variation of architectural and photosynthesis traits had rather small effects on canopy light absorption and net photosynthesis in all planting densities, ranging from +0.3% to −2.2% for the individual traits (**Figure 3**; see **Supporting Information**-**Figure S4** for absolute values on light absorption and net photosynthesis for the reference scenario). When interplant variations of all architectural traits were simultaneously included in the model, the canopy light absorption was 3.1% lower and the net photosynthesis was 2.4% lower than for the simulation without any interplant variation. Adding interplant variation of all photosynthesis traits to interplant variation in all architecture traits further decreased canopy net photosynthesis by −1.1%; hence, net photosynthesis was 3.5% lower when variation in architectural as well as photosynthesis traits was considered compared with a crop without any interplant variation. The coefficient of variation in plant light absorption and net plant photosynthesis was higher than the planting density; for the reference plants (where the random rotation of plants relative to their neighbors was the only source of variation), the coefficient of variation increased from 2.4% to 5.5% for PAR absorption, while the coefficient of variation increased from 2.0% to 5.6% for net photosynthesis when planting density increased from 1.5 to 3.3 plants m^−2^ (**Figure 3**). Introducing interplant variation in internode length and leaf area increased the coefficient of variation of light absorption and net photosynthesis by up to 4.0%.

### Season and fraction direct light have minor effects on canopy light absorption and photosynthesis

**Figure 4:**
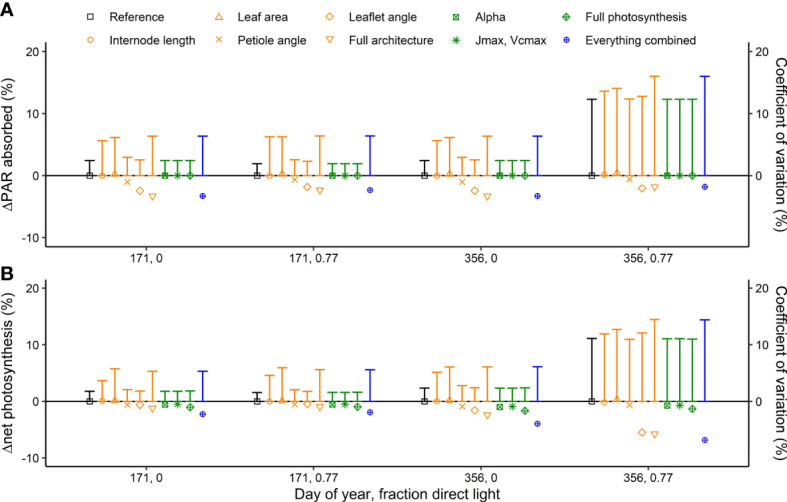
Simulated effects of interplant variation in architectural and photosynthesis traits on **(A)** canopy light absorption (symbols) and the coefficient of variation in plant light absorption (vertical bars) and **(B)** net photosynthesis (symbols) and the coefficient of variation in photosynthesis (vertical bars) at two fractions of direct sunlight (0 and 0.77) and at summer and winter solstices (DOY 171 and 356). Canopy light absorption and net photosynthesis are presented as % change compared to the reference where the rotation of plants relative to their neighbors (0 to 360◦, drawn at random) was the only source of variation. The rotation of the plants relative to their neighbors was kept the same for every model simulation such that any differences in light absorption and net photosynthesis are the result of including interplant variation of architectural and/or photosynthesis traits. Data are the averaged values of all hourly time steps in a day of 24 plants (centre 12 plants of the centre two double rows) from five repetitions (simulations with each a different random number generator for the light model and drawings from the normal distribution) and three different planting densities (1.5, 2.4 and 3.3 plants m^−2^, resulting in a total of 360 plants).

**Figure 5:**
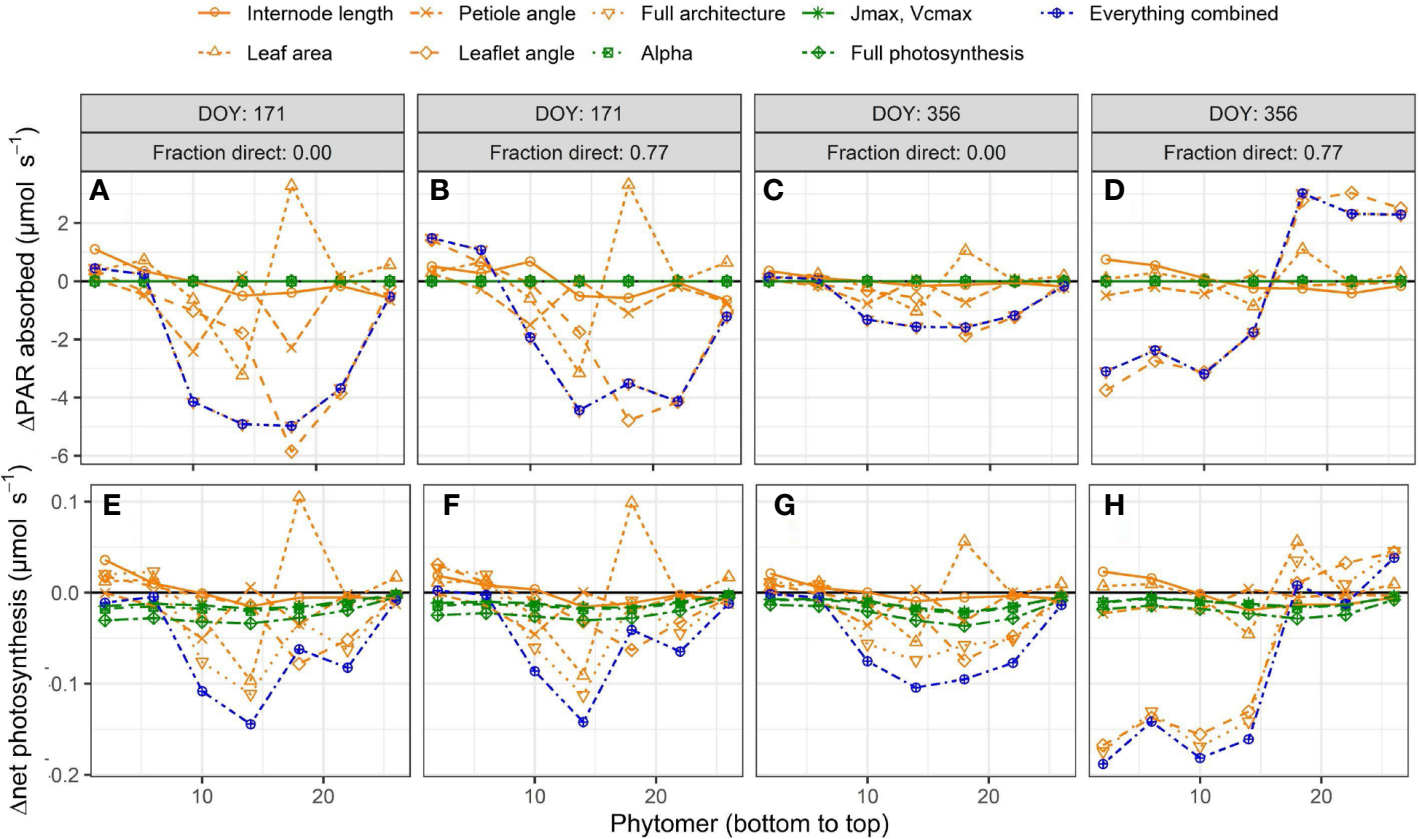
Simulated effects of interplant variation in architectural and photosynthesis traits on differences in leaf light absorption **(A–D)** and net photosynthesis **(E–H)** at a fraction of 0 **(A,C,E,G)** or 0.77 direct sunlight (B,D,F,H) and at summer **(A,B,E,F)** and winter solstices **(C,D,G,H)**. Each simulation is compared to the reference where the rotation of plants relative to their neighbors (0 to 360◦, drawn at random) was the only source of variation. The rotation of the plants relative to their neighbors was kept the same for every model simulation such that any differences in light absorption and net photosynthesis are the result of including interplant variation of architectural and/or photosynthesis traits. Data represent the average of 3 adjacent phytomer ranks (i.e. phytomer rank 2 is the average of phytomer ranks 1 to 3; phytomer rank 6 the average of phytomer ranks 5 to 7) of all hourly time steps in a day from 24 plants (centre 12 plants of the centre two double rows) from five repetitions, at three different planting densities (1.5, 2.4 and 3.3 plants m−2), resulting in a total of 360 observed plants.

Introducing interplant variation in architectural and photosynthetic traits had a limited effect on canopy light absorption (**Figure 4**). Introducing variation in architectural traits together with photosynthetic traits reduced canopy light absorption by up to −3.3% (**Figure 4**). These effects were similar whether it was summer or winter solstice and whether there was 0% or 77% direct light. For canopy net photosynthesis, the effects were the largest on a day with a large fraction (77%) of direct light in winter when net photosynthesis was reduced by 6.8% due to introducing interplant variation in architectural and photosynthetic traits. On this day, there was an increase in canopy light absorption at the upper part of the canopy, while there was a decrease in the lower part of the canopy, resulting in a lower canopy net photosynthesis (**Figure 5**). On days with 77% direct light in winter, the interplant variation in light absorption and net photosynthesis was larger compared with all the other days.

## Discussion

### Interplant variation reduces canopy light absorption and net photosynthesis

The aim of this study was to determine the importance of observed interplant variation in architectural and photosynthetic traits on canopy light absorption and net photosynthesis. We found that introducing interplant variation in architectural and photosynthetic traits in an FSPM did not affect—depending on the trait—or negatively affected canopy light absorption and net photosynthesis compared with the reference model without interplant variation.

By using a fully grown tomato crop in a static FSPM, the traits could be individually explored at relative ease. This was justified as tomato has a near year-round growth season where the majority of the production is in a canopy that is relatively static. This study used a well-established photosynthesis modeling with a plant architecture (e.g., Zhang et al., 2021; van der Meer et al., 2021) to understand the influence of certain architectures resulting from plant variability at a long-lasting growth stage. The same architectural parameters can have varying effects when actual growth is simulated (e.g., Chen et al., 2014), and would result in the observation that during a growth cycle traits may have positive or negative effects depending on the developmental stage in which the research is performed (Falster et al., 2018). By using a static FSPM, we observed that mainly interplant variation in leaflet angles reduces canopy light absorption and photosynthesis, while in particular interplant variation in the leaf area increased the coefficient of variation of plant light absorption and net photosynthesis. This suggests that for the purpose of accurately determining canopy light absorption and net photosynthesis, petiole and leaflet angle should be considered, whereas leaf area should be considered for accurately determining the coefficient of variation in plant light absorption and net photosynthesis. In this study, we investigated the consequences of variation in quite a number of architectural and photosynthetic traits. In real plants, there may even be some more traits that show variation, like irregularities in leaf surface which may affect light interception. Though not investigated here, we may expect a similar response as to the variation in other parameters that determine leaf shape. Previous static tomato FSPM reported values of differences in canopy net photosynthesis up to 7% for simulations when the leaflet angle was changed (Sarlikioti et al., 2011a; van der Meer et al., 2021). Values in this study were within the same range, although in this case interplant variation in architectural and photosynthesis parameters was introduced instead of a sensitivity analysis. Applying interplant variation in photosynthetic traits reduced canopy net photosynthesis. The negative effects on net photosynthesis can be explained by the non-linear relationship between alpha, *J*_max_, and V_cmax_ with photosynthesis (Wilson et al., 1992; Sarlikioti et al., 2011b; Zhu et al., 2012; Song et al., 2013; Von Caemmerer, 2013). The observation of a negative effect on average net photosynthesis when light is redistributed toward the top of the canopy is shown by Vermeiren et al. (2020) and observed in this study on a winter solstice with 77% direct light (**Figures 5D, H**). A higher fraction of light absorption by the top of the canopy is critical at the young stage to increase light absorption for canopy establishment (Richter et al., 2010) and can have stacking effects, as observed by Chen et al. (2014) with increases in biomass production up to 20%.

### Increased planting density increases the coefficient of variation in plant light absorption and net photosynthesis without affecting average net photosynthesis

Higher planting density increased the coefficient of variation in plant light absorption and net photosynthesis without affecting overall canopy photosynthesis. This is related to an increase in light competition within the canopy at higher planting density. For every architectural and photosynthesis parameter included in this study, we have shown that introducing interplant variation in leaf area results in the highest coefficient of variation in plant light absorption and net photosynthesis.

### Implications for functional–structural plant modeling of crop production

Incorporating realistic variation of all architectural and photosynthesis traits in the model reduced daily canopy light absorption by 1.8% to 3.3% and daily canopy net photosynthesis by −1.9% to −6.8%, with the largest differences in winter when the solar angle is relatively low.

In Experiment 2, the measured coefficient of variation in fruit production per plant was on average 12.8%, while in our model simulation, the coefficient of variation for whole-plant net photosynthesis reached values up to 12.3%, although generally lower than 8%. Our model might not yet be able to capture all interplant variability, as variation in reality may also arise from variation in local (a)biotic factors which were not considered in our study.

Our data were based on tomato grown on a substrate (stonewool) in modern high-tech greenhouses. In these systems, usually homogeneous hybrid plants are used and the variability in climate and conditions in the rooting zone is relatively low. Furthermore, growers apply quite some plant training (tying to the strings for vertical growth, pruning of side shoots, pruning of leaves and fruits), which reduces heterogeneity in the canopy. In crops that are less managed, the variation will likely be larger than in our study. In field crops where plants are grown in the soil, the variability further increases due to variations in soil conditions.

In this study, we used a static model to estimate interplant variation. In a dynamic model, it is likely that an initial variation among plants will increase over time as taller plants may shade lower plants, and therefore, these taller plants get rapidly larger than the other plants.

## Conclusions

Depending on the trait, introducing interplant variation in architecture and photosynthesis traits in a functional–structural plant model did not affect or negatively affected canopy light absorption and net photosynthesis compared with the reference model without interplant variation. Considering the realistic variation of all architectural and photosynthesis traits reduced daily canopy light absorption by −1.8% to −3.3% and daily canopy net photosynthesis by 1.9% to 6.8%, with the largest differences in winter when solar angle is relatively low. Introducing interplant variation of architectural and photosynthesis traits in FSPM results in a more realistic simulation of variation of plants within a canopy. Furthermore, it can improve the accuracy of simulation of canopy light interception and photosynthesis, though these effects at the canopy level are relatively small (*<*4% for light absorption and<7% for net photosynthesis).

## Data availability statement

The raw data supporting the conclusions of this article will be made available by the authors, without undue reservation.

## Author contributions

MM, PV, EH, and LM devised the research and the main conceptual ideas. MvdM designed the simulation scenarios together with HL, PV, and LM. MM adapted the functional–structural plant model and executed the simulations together with HL. MM processed the data together with HL. All authors were involved in data analysis. MvdM drafted the manuscript and designed the figures. LM, EH, and PV supervised the research, and they edited the manuscript. All authors contributed to the article and approved the submitted version.

## Supplementary material

Supplementary Table 1 Values for the dimensionless parameters *A* and *B* of Eq. 1 for calculating leaf area of Exp. 1 according to an empirical relationship acquired from data in Exp. 1.

Supplementary Table 2 Composition of the nutrient solution used in the experiment. Solution EC: 2.8 and pH: 5.5.

Supplementary Table 3 Testing assumptions made in ray number and GroIMP’s setseed (random number generator) on canopy light absorption and net photosynthesis on two simulated days of the year (DOY).

Supplementary Table 4 Absolute values and standard deviation of light absorbed and net photosynthesis per m^2^ floor area for model simulations with the reference scenario on different days of the year (171, 356), planting densities (1.5, 2.4 and 3.3) and fractions of direct sunlight (0 and 0.77). Simulation results were averaged over planting density for the first four rows, and averaged over the combination of day of year (DOY) and fraction direct light for the last three rows. This was done as no clear interaction effects were observed was observed from the individual plots differing these individually.

Supplementary Figure 1 Example of implementing interplant variation for trait X. To keep the total trait value equal across the canopy as a whole a value is taken from the normal distribution and a mirrored value is taken for another plant in the canopy.

Supplementary Figure 2 Simulated effects of interplant variation in architectural and photosynthesis traits on absolute differences in leaf light absorption and net photosynthesis (in *µ*mol s*^−^* ^1^) at three planting densities (1.5, 2.4 and 3.3 plants m*^−^* ^2^). Each simulation is compared to the reference where the rotation of plants relative to their neighbors (0 to 360°, drawn at random) was the only source of variation. The rotation of the plants relative to their neighbors was kept the same for every model simulation such that any differences in light absorption and net photosynthesis are the result of including interplant variation of architectural and/or photosynthesis traits. Data represent the average of 3 adjacent phytomer ranks (i.e. phytomer rank 2 is the average of phytomer ranks 1 to 3; phytomer rank 6 the average of phytomer ranks 5 to 7) of all hourly time steps in a day from 24 plants (centre 12 plants of the centre two double rows) from five repetitions, at two fractions of direct light (0 and 0.77) and two solstices summer and winter (DOY 171 and 356), resulting in a total of 480 observed plants.
