# Supplementary material for: Consequences of interplant trait variation for canopy light absorption and photosynthesis
Source: Front Plant Sci. 2023 Jan 20;14:1012718. doi: 10.3389/fpls.2023.1012718 (PMC9895853; doi:10.3389/fpls.2023.1012718)
Supplement: Supplementary Table 1 — Values for the dimensionless parameters A and B of Eq. 1 for calculating leaf area of Exp. 1 according to an empirical relationship acquired from data in Exp. 1. [file DataSheet_1.docx]

# Consequences of interplant trait variation for canopy light absorption and photosynthesis

van der Meer, M. ^1^, Lee, H. ^1^, de Visser, P. H. B. ^2^, Heuvelink, E. ^1^, Marcelis, L. F. M. ^1#^

# SUPPLEMENTARY MATERIAL

Table S1: Values for the dimensionless parameters *A* and *B* of Eq. [1](#_bookmark14) for calculating leaf area of Exp. 1 according to an empirical relationship acquired from data in Exp. 1.

| Cultivar | *A* | *B* |
| --- | --- | --- |
| Foundation | 0.2533 | 1.0125 |
| Progression | 0.2389 | 1.0164 |
| Extension | 0.2257 | 1.0350 |
| 9112 | 0.1963 | 1.0624 |

Table S2: Composition of the nutrient solution used in the experiment. Solution EC: 2.8 and pH: 5.5.

| Macronutrients | Amount | Unit | Micronutrients | Amount | Unit |
| --- | --- | --- | --- | --- | --- |
| NH4 | 1.2 | mmol/l | Si | 0 | *µ*mol/l |
| K | 10.3 | mmol/l | Fe | 25 | *µ*mol/l |
| Na | 0 | mmol/l | Mn | 10 | *µ*mol/l |
| Ca | 5.8 | mmol/l | Zn | 5 | *µ*mol/l |
| Mg | 2.59 | mmol/l | B | 30 | *µ*mol/l |
| NO3 | 17.2 | mmol/l | Cu | 0.75 | *µ*mol/l |
| SO4 | 4.76 | mmol/l | Mo | 0.5 | *µ*mol/l |
| HCO3 | 0 | mmol/l | Cl | 0 | *µ*mol/l |
| P | 1.62 | mmol/l |  |  |  |

Table S3: Testing assumptions made in ray number and GroIMP’s setseed (random number generator) on canopy light absorption and net photosynthesis on two simulated days of the year (DOY).

| Light absorbed | | | | Net photosynthesis |
| --- | --- | --- | --- | --- |
| DOY | milRays | setSeed  (*µ*mol m*^−^*^2^ s*^−^*^1^) | | (*µ*mol m*^−^*^2^ s*^−^*^1^) |
| 171 | 750 | 5 | 1307.8 ±24.8 | 7.91 ±0.07 |
| 171 | 1250 | 5 | 1307.7 ±24.8 | 7.91 ±0.07 |
| 171 | 750 | 6 | 1307.7 ±24.6 | 7.91 ±0.07 |
| 171 | 1250 | 6 | 1307.5 ±24.8 | 7.91 ±0.07 |
| 356 | 750 | 5 | 601.8 ±61.7 | 5.70 ±0.37 |
| 356 | 1250 | 5 | 601.8 ±61.7 | 5.70 ±0.37 |
| 356 | 750 | 6 | 601.8 ±61.6 | 5.71 ±0.37 |
| 356 | 1250 | 6 | 601.8 ±61.6 | 5.71 ±0.37 |

Table S4: Absolute values and standard deviation of light absorbed and net photosynthesis per m^2^ floor area for model simulations with the reference scenario on different days of the year (171, 356), planting densities (1.5, 2.4 and 3.3) and fractions of direct sunlight (0 and 0.77). Simulation results were averaged over planting density for the first four rows, and averaged over the combination of day of year (DOY) and fraction direct light for the last three rows. This was done as no clear interaction effects were observed was observed from the individual plots differing these individually.

| DOY Plant Density Fraction Light absorbed | Net photosynthesis |
| --- | --- |

|  | (plants m*^−^*^2^) | direct light | (*µ*mol m*^−^*^2^ s*^−^*^1^) | (*µ*mol m*^−^*^2^ s*^−^*^1^) |
| --- | --- | --- | --- | --- |
| 171 | 1.5, 2.4, 3.3 | 0 | 1132.1 ±27.5 | 40.85 ±0.73 |
| 171 | 1.5, 2.4, 3.3 | 0.77 | 1098.4 ±21.2 | 36.80 ±0.58 |
| 356 | 1.5, 2.4, 3.3 | 0 | 361.4 ±8.8 | 19.93 ±0.47 |
| 356 | 1.5, 2.4, 3.3 | 0.77 | 461.8 ±56.9 | 22.80 ±2.54 |
| 171, 356 | 1.5 | 0, 0.77 | 697.4 ±16.5 | 24.97 ±0.51 |
| 171, 356 | 2.4 | 0, 0.77 | 771.3 ±32.8 | 30.90 ±1.30 |
| 171, 356 | 3.3 | 0, 0.77 | 821.5 ±45.2 | 34.41 ±1.92 |


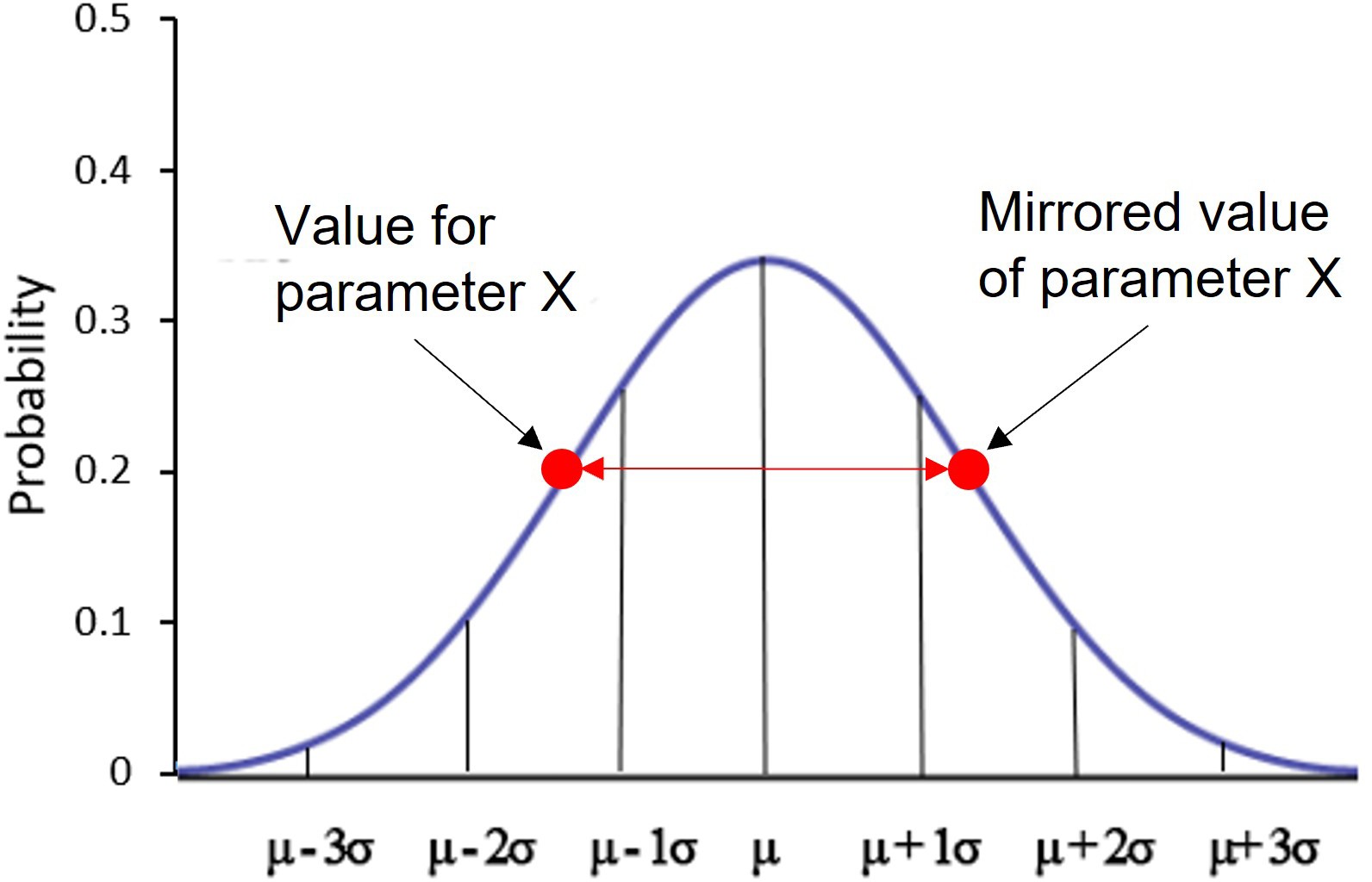


Figure S1: Example of implementing interplant variation for trait X. To keep the total trait value equal across the canopy as a whole a value is taken from the normal distribution and a mirrored value is taken for another plant in the canopy.


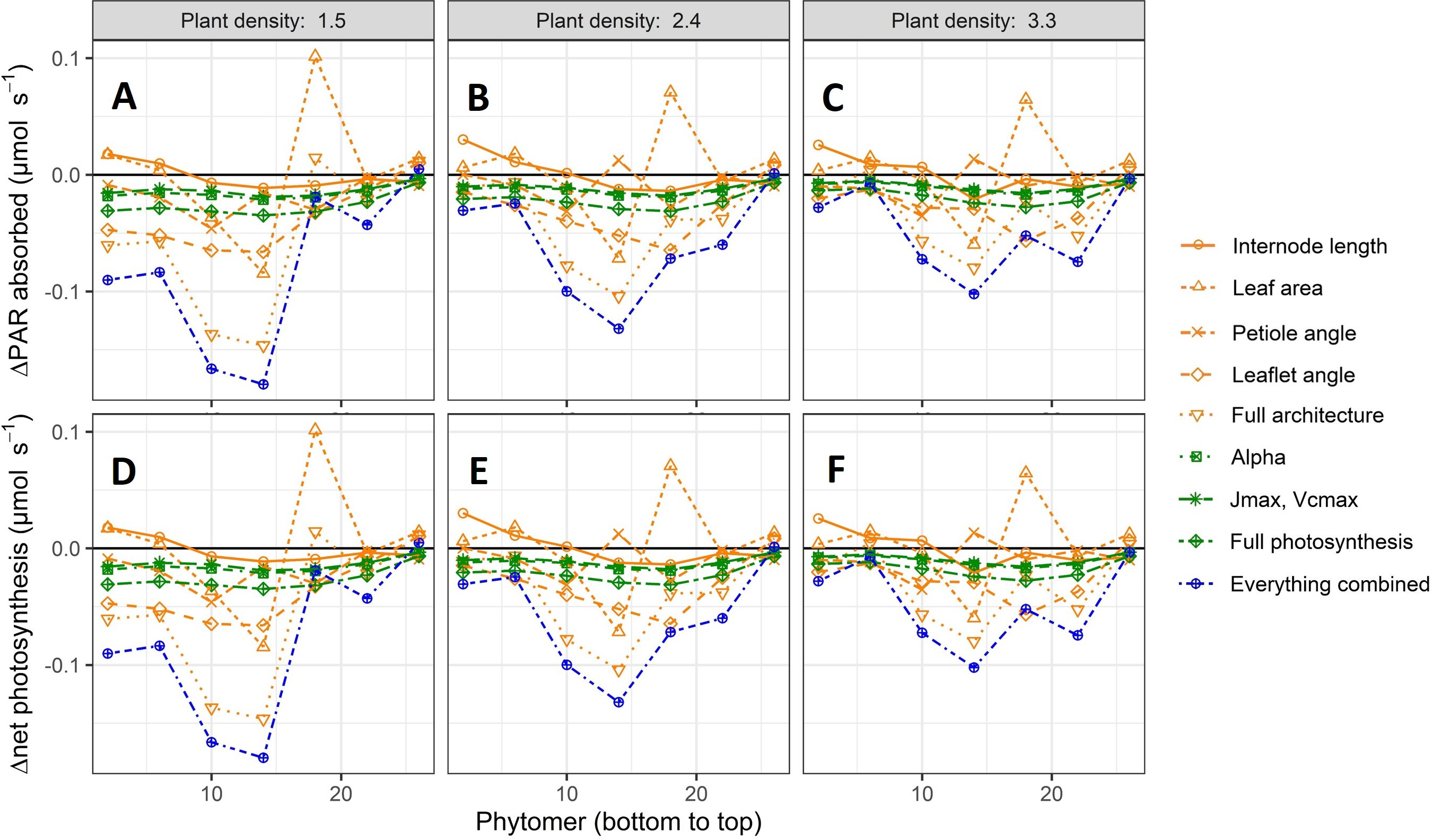


Figure S2: Simulated effects of interplant variation in architectural and photosynthesis traits on absolute differences in leaf light absorption (A,B, C) and net photosynthesis (in *µ*mol s*^−^*^1^; D,E,F) at three planting densities (1.5, 2.4 and 3.3 plants m*^−^*^2^). Each simulation is compared to the reference where the rotation of plants relative to their neighbors (0 to 360*^◦^*, drawn at random) was the only source of variation. The rotation of the plants relative to their neighbors was kept the same for every model simulation such that any differences in light absorption and net photosynthesis are the result of including interplant variation of architectural and/or photosynthesis traits. Data represent the average of 3 adjacent phytomer ranks (i.e. phytomer rank 2 is the average of phytomer ranks 1 to 3; phytomer rank 6 the average of phytomer ranks 5 to 7) of all hourly time steps in a day from 24 plants (centre 12 plants of the centre two double rows) from five repetitions, at two fractions of direct light (0 and 0.77) and two solstices summer and winter (DOY 171 and 356), resulting in a total of 480 observed plants.
